# Book Review: Representations of the World in Language Textbooks

**DOI:** 10.3389/fpsyg.2022.860465

**Published:** 2022-03-11

**Authors:** Mengmeng Bo

**Affiliations:** Faculty of Education, Shanghai Normal University, Shanghai, China

**Keywords:** representation, language textbooks, culture learning, culture analysis, second/foreign language pedagogy

Given its interrelation with culture, language is perceived as a symbolic representation of people (Mahmoud, 2015). Risager's (2021) book *Representations of the World in Language Textbooks* seeks to find out images of culture, society, and the world in language pedagogy by analyzing foreign language textbooks in six languages (English, German, French, Spanish, Danish, and Esperanto). Risager has made a remarkable contribution in breaking through the gap between theory and practice. His empirical analysis of culture in textbooks distinguishes five theoretical approaches: national studies, citizenship education studies, Cultural studies, postcolonial studies, and transnational studies.

Chapter 1, *Representations of the World*, focuses on the perspectival nature of knowledge representation. The author uses Foucault's explanation of knowledge: “it is socially situated and embedded in discourses, always seen, and represented from somewhere and by some people with life histories, experiences, and power positions” (Foucault, 1976). Furthermore, the chapter discusses three approaches to representation: the reflective, the intentional and the constructionist. The reflective approach implies that representation directly reflects reality; the intentional approach suggests the intention of the textbook writer plays a visible role in representation. The constructionist approach advocates those individual readers construct dynamic representations of the textbook. In the following, the author mainly illustrates the characteristics of the five theoretical approaches upon which textbooks are analyzed, discusses the role of publishers and the selection of textbooks, then introduces the Danish education system.

Chapter 2, *Culture in Textbooks Analyses around the World*, begins with a presentation of three kinds of methodologies in the field of textbook analysis: thematic analysis, intercultural analysis, and power and empower analysis. Thematic analysis regards knowledge as factual knowledge that can be divided into various themes and topics. Intercultural analysis implies the construction of knowledge resides in “students' awareness of different sociocultural perspectives, identities and their implications for intercultural communication and understanding, empathy and collaboration” (p. 28). Power and empower analysis favor the method of critical discourse analysis, seeing the representations of world as conflicts, ideologies, and intercultural learning. In order to concretely illustrate the three methodologies, the author discusses related theoretical results in the sections that focus on thematic categories, including different parts of the world, geographical contexts, genres of textbooks and cultural politics. The last part of this chapter introduces a survey corpus and advice that people working with different languages should learn from each other.

Based on the English language textbook *A Piece of Cake* used in Denmark and the German language textbook *Du bist dran*, Chapter 3 discusses the analysis approach of “national studies”, a term coined by the author who believes there are two central concepts: banal nationalism and the distinction between political and ethnic conceptions of the national (Risager, 2007). The former refers to “the patterns of belief and practice which reproduce the world…, in which ‘we' live as citizens of nation-states” (Billig, 1995). The latter is described by Smith (1986) as the political national model based on a common political, legal, economic system, a common culture, religion, and language, whereas the ethnic national model deems the first language as identity factors. The author employs the critical discourse analysis method to discuss the positioning and representation of actors, representation of culture, society and the world, and intercultural learning approach. It is found that even though both textbooks express nationalistic sentiments, they fail to represent national identity and do not adopt a global and transnational perspective. Furthermore, there is an unbalanced representation of the culture of target language countries. With regards to intercultural study, both textbooks comprise the culture comparison but do not show the real intercultural settings nor examples of intercultural communication strategies. Lastly, the author suggests textbook writers should consider which language (students' first language or foreign language) is more effective in the process of knowledge construction.

Chapter 4 focuses on citizenship education. First, the chapter defines the concept, highlights its underpinning theory, and explores its relationship with pedagogy. Regarding citizenship education studies in language and culture pedagogy, the author mainly concentrates on Michael Byram's concepts of “intercultural citizenship” and “critical cultural awareness”. Then, the author selects the chapter on “The Environment” in *A Piece of Cake* and the chapter on “Miljø (the environment)” in *Puls* (Danish texbooks) as a citizenship education analysis sample. The analysis indicates these two series of textbooks seldom include any discussions on political system and ideology, although the freedom to discuss political issues in Denmark is relatively high. Furthermore, neither of the textbooks regards target language countries as societies with diverse cultures, languages, and beliefs, nor integrates citizenship education into teaching materials. Meanwhile, the characteristics of language used in these textbooks reflect the language status struggle between English and Danish in Denmark.

Chapter 5 centers on Cultural studies. The chapter begins with a review of the development of Cultural studies, interprets the main views of Stuart Hall, and briefly summarizes the contents of current Cultural studies. The author then puts forward the concept of linguaculture that integrates three different dimensions of language: the semantic and pragmatic dimension, the poetic dimension, and the identity dimension, followed by a discussion of Kramsch, Holliday, Guilherme, and Gray's opinions of Cultural studies in language and culture pedagogy, The author continues to explore the analysis framework proposed in Chapter 1 to analyze the theme of “Teen issues” in *A Piece of Cake* and “Être différent” in *Francais Formidable* (French textbooks). The author concludes that while these two types of textbooks are related to race, religion, and social class, they pay little attention to gender issues.

Chapter 6 dedicated to *Postcolonial Studies*, introduces the concept outlines its development and relationship with language teaching. As for postcolonial studies in language and culture pedagogy, the author explains the concept of “the worldliness of English” (Pennycook, 1994), which is “a way of foregrounding the connections between languages and its social, economic, cultural, political and historical contingencies” (p. 179). After comparing *A Piece of Cake* with *Caminando* (Spanish textbooks), the author concludes that *A Piece of Cake* presents the postcolonial history from a Caucasian perspective; although *Caminando* contains postcolonial knowledge, it adopts a European centric position and makes no mention of the social and historical impacts of colonialism; both of them “only offer students minute inputs to support and enlarge their knowledge about the historical spread of English and Spanish, and about the world in terms of historically developed global power relations and in terms of racial hierarchies” (p. 203–204).

The last chapter discusses transnational studies, defining the concept as “an optic or gaze that begins with a world without borders” (Khagram and Levitt, 2008). Also, the author illustrates Appadurai's (1996) theory of five kinds of cultural flow from a transnational angle, namely, ethnoscape, mediascape, technoscape, financescape, and ideoscape. In terms of translational studies in language and culture, the author believes textbooks should not only be limited to the national paradigm, given the primary purpose of language education is to enhance students' global cultural awareness. Therefore, the author analyzes transnational culture that is represented in *A Piece of Cake* and *Vojago* (Esperanto textbooks). The analysis indicates that both textbooks present the transnational culture in different ways. *A Piece of Cake* has only one unit entitled “globalization”, which focuses on the culture of globalization and the relationship among English-speaking countries. For their part, Esperanto textbooks guide learners to observe the world through the “Esperanto club”, highlighting the mobility of Esperanto users and the significance of international Esperanto organizations.

Finally, Risager concludes that although the six language textbooks deftly present factual knowledge, epistemological knowledge, and common sense, almost every knowledge construction pattern relies on the stacking of facts, which neglects the critical awareness of identifying different ideas, identities, and epistemologies. Besides, based on the five analyzing approaches, the author expands the connotation of intercultural competence (IC). At the end of this volume, Risager emphasizes the dual focus of language textbooks: “on the one hand, language and language learning, on the other hand, culture, society, and the world and intercultural learning” (p. 248).

All in all, this book presents an innovatively comprehensive framework to analyze representations of culture, society, and the world in foreign and second language learning materials. This volume's findings and suggestions logically align with each theoretically informed analysis susceptible to practically benefit global teaching professionals, teaching/learning materials writers, publishers, and second/foreign language teaching researchers. Given the current interest in establishing connections among language, history, politics, and sociology, the book is a timely contribution, particularly for multi-disciplinary researchers looking into this domain.

Theoretically, this book reinforces the statement language teaching involves culture teaching (Allwright and Bailey, 1991), and the five theoretical approaches of national studies, citizenship education studies, Cultural studies, postcolonial studies, and transnational studies offer both a theoretical framework and perspective for language textbooks analysis. Conceptually, the book revisits the concept of linguaculture and re-establishes the connotation of IC. Methodologically, the five approaches clarify the cultural dimensions of language learning and they are empirically relevant for multi-disciplinary textbooks analysis.

With all the merits mentioned, readers may find it even more comprehensive if it had included more discussions about Asian language textbooks, including Chinese or Japanese, likely to provoke further reflection on the cultural representations between the East and the West in a globalized context.

## Author Contributions

The author confirms being the sole contributor of this work and has approved it for publication.

## Conflict of Interest

The author declares that the research was conducted in the absence of any commercial or financial relationships that could be construed as a potential conflict of interest.

## Publisher's Note

All claims expressed in this article are solely those of the authors and do not necessarily represent those of their affiliated organizations, or those of the publisher, the editors and the reviewers. Any product that may be evaluated in this article, or claim that may be made by its manufacturer, is not guaranteed or endorsed by the publisher.

## References

[B1] Allwright D. Bailey K. M. (1991). Focus on the Language Classroom: an Introduction to Classroom Research for Language Teachers. Cambridge: Cambridege University Press.

[B2] Appadurai A. (1996). “Modernity at Large,” in Cultural Dimensions of Globalization. Minneappolis MN: University of Minnesota Press.

[B3] Billig M. (1995). Banal Nationalism. London: Sage.

[B4] Foucault M. (1976). “La volonté de savoir,” Historie de la Sexualité 1. Paris: Gallimard.

[B5] Khagram S. Levitt P. (2008). “Constructing Transnational Studies,” in The Transnational Studies Reader: Intersections and Innovations, eds. D. H. Khagram and P. Levitt. New York/London: Routledge. p. 1–19.

[B6] Mahmoud M. A. A. (2015). Culture and English language teaching in the Arab world. Adult Learning. 26, 2. 10.1177/1045159515573020

[B7] Pennycook A. (1994). The Cultural Politics of English as an International Language. New York: Longman.

[B8] Risager K. (2007). Language and Culture Pedagogy: from a National to a Transnational Paradigm. Clevedon: Multilingual Matters. 10.21832/9781853599613

[B9] Risager K. (2021). “Representations of the world in language textbooks,” in Languages for Intercultural Communication and Education (Beijing: Foreign Language Teaching and Research Press), 288 p.

[B10] Smith A. D. (1986). The Ethnic Origins of Nations. London: Blackwell.

